# Development of a molecular genetics and cell biology toolbox for the filamentous fungus *Diplodia sapinea*

**DOI:** 10.1371/journal.pone.0308794

**Published:** 2024-12-27

**Authors:** Anne Geertje Oostlander, Laura Brodde, Miriam von Bargen, Bernard Slippers, Yvonne Becker, Ulrike Brandt, Frank Klawonn, Christiaan Grobler, Lucas Well, Jan Stenlid, Jonas Oliva, Malin Elfstrand, Andre Fleissner

**Affiliations:** 1 Institute of Genetics, Technische Universität Braunschweig, Braunschweig, Germany; 2 Department of Forest Mycology and Plant Pathology, Swedish University of Agricultural Sciences, Uppsala, Sweden; 3 Department of Biochemistry, Genetics and Microbiology, Forestry and Agricultural Biotechnology Institute (FABI), University of Pretoria, Pretoria, South Africa; 4 Institute for Epidemiology and Pathogen Diagnostics, Julius Kühn Institute (JKI)—Federal Research Centre for Cultivated Plants, Braunschweig, Germany; 5 Helmholtz Centre for Infection Research (HZI), Braunschweig, Germany; University of Salento Department of Biological and Environmental Sciences and Technologies: Universita del Salento Dipartimento di Scienze e Tecnologie Biologiche ed Ambientali, ITALY

## Abstract

*Diplodia sapinea* (Fr.) Fuckel is a widespread fungal pathogen affecting conifers worldwide. Infections can lead to severe symptoms, such as shoot blight, canker, tree death, or blue stain in harvested wood, especially in *Pinus* species. Its impact on forest health is currently intensified, likely due to climate change, posing an increasing threat to global ecosystems and forestry. Despite extensive and successful research on this pathogen system, fundamental questions about its biology and plant-associated lifestyle remain unanswered. Addressing these questions will necessitate the development of additional experimental tools, including protocols for molecular genetics and cell biology approaches. In this study, we continue to address this need by establishing an *Agrobacterium*-mediated genetic transformation protocol for *D*. *sapinea*, enabling targeted mutagenesis and heterologous gene expression. We utilized this methodology to localize the histone H2B by tagging it with the fluorescent protein mCherry. Additionally, we established a time- and space-efficient laboratory-scale infection assay using two-week-old *Pinus sylvestris* seedlings. Integrating these tools in a proof-of-concept study enabled the visualization of *D*. *sapinea in planta* growth through the fluorescently labeled reporter strain.

## Introduction

*Diplodia sapinea* (Fr.) Fuckel (syn. *Diplodia pinea* (Desm.) Kickx., *Sphaeropsis sapinea* (Fr.: Fr.) Dyko & Sutton) is a globally distributed opportunistic pathogen of conifers, capable of causing significant losses in various species, including economically important ones such as *Pinus sylvestris* and *Pinus radiata* [1]. In recent years, disease incidences have surged, likely due to heightened host plant stress from escalating extreme weather and climate conditions [2, 3]. Disease symptoms caused by *D*. *sapinea* include shoot blight, canker, needle discoloration, root disease, limited growth and even tree death [2, 4]. The fungal infection is also known to cause extensive blue-stain in harvested wood, reducing the market value of the wood [5]. The cumulative damages inflicted by *D*. *sapinea* result in substantial economic losses to forest companies [6–8]. The global occurrence and impact of *D*. *sapinea* is on the rise, with its distribution shifting northward, largely attributed to climate warming, especially noticeable in Europe [9–11]. This widespread occurrence and high infestation levels of *D*. *sapinea* in affected areas make it one of the most significant pine pathogens globally, contributing to the decline of critical forest ecosystems [12].

Despite over 150 years of research on *D*. *sapinea*, numerous questions persist about its biology and infection processes, including its potential for latent pathogenicity and/or endophytic growth. The pathogen can infect pine species without triggering a host response and persist in the plant for extended periods, even decades [13, 14]. The fungus has been reported in various healthy plant tissues, including roots, stems, shoots, cones, buds and needles of various *Pinus* species [13, 15–18]. A current hypothesis suggests that this symptomless presence in the plant provides the basis for symptom-causing growth of *D*. *sapinea*, triggered by plant stressors like mechanical wounding, drought, or heat [19–21]. Open questions include the molecular and cellular mechanisms mediating stress induced disease onset, tissue penetration, *in planta* growth, evasion of host recognition, and long-term persistence.

Addressing these questions requires expanding the methodological repertoire for *D*. *sapinea* research. Existing methods and resources include isolation from plant tissues, or pycnidia found on pine cones or other tissues, markers for population genetics, species identification and studying asymptomatic infections, and a robust taxonomy, with an epitype culture and genome sequence [1, 22–24]. Pathogenicity studies often use wounding and mycelial plug inoculation, which may not mimic natural infection [25–27]. Sporulation has traditionally been induced on water agar containing pine needles, but this method non-standardized, labor-intensive, and yields limited pycnidia [28, 29]. In a recent study, we enhanced sporulation induction and spore-based plant inoculation [30], offering a more robust approach that better mimics natural infection.

Tools for molecular and cell biological analysis for *D*. *sapinea*, such as functional genetics, remain very limited. For reverse genetics approaches, transformation protocols are required. Several transformation methods have been established for a broad range of filamentous fungi, including PEG-mediated protoplast transformation, electroporation, biolistic transformation, and *Agrobacterium*-mediated transformation. However, these methods are not universally applicable, requiring adaptation for each individual species [31, 32].

*Agrobacterium*-mediated transformation stands out by eliminating the need for laborious protoplast generation or specialized technical equipment, therefore offering simplicity [33, 34]. Furthermore, it demonstrates a high success rate, even in fungal species where other transformation methods have proven ineffective [34, 35]. It efficiently produces a high percentage of transformants with single-copy integrated DNA and also facilitates homologous recombination, required for targeted mutagenesis [36]. Species successfully transformed by *Agrobacterium* include *Botryosphaeria dothidea* and *Botryosphaeria kuwatsukai*, both belonging to the same family, *Botryosphaeriaceae*, as *D*. *sapinea* [37, 38].

To understand cellular processes during infection, advanced microscopy methods, like fluorescence protein labeling, laser scanning microscopy, and live cell imaging, are crucial. These techniques allow the visualization of the interplay between fungi and plants, therefore providing the basis for functional gene analysis, for example by gene knockout mutant characterization. The immense power of such combined approaches has been demonstrated in well-established model organisms for fungal plant pathology, such as the hemibiotrophic rice blast fungus *Magnaporthe oryzae* and the grass endophyte *Epichloe festucae* [39–41].

In this study, we built on our previously established spore production and infection protocol [30] and devised a transformation technique for gene targeting and protein fluorescence labeling. Additionally, we developed a space- and time-efficient infection assay using young *P*. *sylvestris* seedlings, enabling laboratory experiments without the need for greenhouse access. By combining these infection, transformation, and genetic manipulation tools, we visualized, in a proof of principle experiment, growth of *D*. *sapinea in planta* using a fluorescence-labeled mutant strain.

We believe that the newly developed tools and protocols will accelerate the pace of *D*. *sapinea* research and facilitate the integration of molecular and cellular biology with field ecology and epidemiology of this fungus, ultimately leading to better strategies to protect Pine forests worldwide.

## Materials and methods

### Strains and growth conditions

The *D*. *sapinea* ex-type strain (CMW 39103/CBS 138184, Table 1) was obtained from Westerdijk Fungal Biodiversity Institute, Utrecht, the Netherlands. The genome sequence of this strain is accessible in GenBank (Genome assembly ASM72994v1; BioProject PRJNA242796).

**Table 1:** Organisms used in this study.

**Fungal strains**			
**Strain number**	**Species**	**Genotype**	**Origin**
GD1_02/ CBS138184	*D*. *sapinea*	Wild type	CBS-KNAW (South Africa, Wubetu Bihon)
GD1_14	*D*. *sapinea*	*niaD*::*hph*:*Ptef-h2b-mCherry-Ttef*	This study
**Bacterial strains**			
EHA105	*Agrobacterium* sp. EHA105	C58 (RifR) Ti pEHA105 (pTiBo542DT-DNA) (GentR), succinamopine	[43]
EHA105_AO11	*Agrobacterium* sp. EHA105	C58 (RifR) Ti pEHA105 (pTiBo542DT-DNA) (GentR), succinamopine, pAO-011, kanR	This study
AGL-1	*Agrobacterium* sp. AGL-1	C58, RecA, pEHA105 (pTiBo542DT-DNA), succinamopine, rifR, carbR	[44]
AGL-1_AO11	*Agrobacterium* sp. AGL-1	C58, RecA, pEHA105 (pTiBo542DT-DNA), succinamopine, pAO-011, rifR, carbR, kanR	This study

Cultivation and harvesting of spores were done as described in Oostlander et al. (2023) [30]. In brief, *D*. *sapinea* was inoculated onto either 120 mm square or 60 mm round Petri dishes containing Vogel’s Minimal Medium (VMM, S1 File) [42] and incubated for at least 21 days under cold daylight (Osram, Lumilux, L 18W/865, Cool Daylight) at 22°C. Conidia were harvested by washing the cultures with sterile 0.01% Tween 20. Spores were washed twice by centrifuging (13 000 rpm, 1 min) and resuspending in 0.01% Tween 20 to remove any traces of liquids washed off the culture.

### Transformation

The transformation protocol described in this peer-reviewed article is published on protocols.io, http://dx.doi.org/10.17504/protocols.io.5qpvok7ozl4o/v1 and is included for printing as S1 File with this article.

#### Plasmid design

The NEBuilder (NEBuilder^®^ HiFi DNA Assembly) cloning strategy was used for the cloning of pAO-011. Fragments for constructing pAO-011 were amplified by polymerase-chain reaction (PCR). The backbone and hygromycin B phosphotransferase gene *hph* under the control of the multifunctional tryptophan biosynthesis protein *trpC* promoter and terminator from *Aspergillus nidulans* were amplified from the plasmid pRFHUE-eGFP [45] with oligonucleotide pairs 2080/2081 and 2084/2085 (S1 Table). The expression cassette additionally carried the sequence encoding a histone *h2b-mCherry* fusion protein under the control of the elongation factor 1 *tef1* promoter and terminator from *D*. *sapinea*. For the construction of the vector, the *h2b* gene of *D*. *sapinea* was amplified by PCR from genomic DNA of the wild-type strain (GD1_02) with the oligonucleotides 2094/2095 (S1 Table). For the *tef* promotor and terminator sequence, 1000 bp upstream and downstream of the *tef* gene of *D*. *sapinea* were amplified by PCR from genomic DNA of the wild-type strain with the oligonucleotide pairs 2096/2097 and 2090/2091 (S1 Table). The expression cassette is flanked by 1000 bp-long sequences from the 3’-UTR and 5’-UTR of the nitrate reductase gene *niaD*, amplified by PCR with the oligonucleotide pairs 2082/2083 and 2086/2087 (S1 Table) from genomic DNA of the wild-type strain. To reduce the number of fragments assembled per reaction, the backbone, *hph* cassette, and *niaD* flanking sequences were initially assembled into the plasmid pAO-010 using the NEBuilder Assembly Cloning Kit. The plasmid was amplified in *E*. *coli*, and a linear fragment from pAO-010 was produced via PCR using the plasmid DNA as a template with the oligonucleotides 2088/2089. This fragment was assembled with the *mCherry*, *h2b*, *Ptef*, and *Ttef* sequences to form the plasmid pAO-011. A test digestion with the restriction enzyme Eco321 followed by gel electrophoresis was conducted to verify the correct assembly of the plasmid.

#### Transformation of electrocompetent *Agrobacterium* sp

Fifty μl of electrocompetent *Agrobacterium* sp. AGL-1 or EHA105 cells [46] were mixed with 1–1.5 μl (150–300 ng) of plasmid DNA (pAO-011) and incubated on ice for 2 min. The DNA-cell mixture was transferred to a 2 mm electroporation cuvette, and electroporated at 2500 V, 25 μF and 400 Ω. After electroporation, cells were incubated at 28°C and 250 rpm for 3 h in LB for recovery. Cells were then spread on LB selective plates (kanamycin 50 μg/ml, rifampicin 25 μg/ml and carbenicillin 100 μg/ml for strain AGL-1 or kanamycin 50 μg/ml and rifampicin 15 μg/ml for strain EHA105) and incubated at 28°C in darkness for 24–72 h. Verification of the transformation was done via colony-PCR. Stocks of the transformed *Agrobacterium* sp. strains were kept in 20% glycerol at -80°C (AGL-1_AO11 and EHA105_AO11).

#### Preparation of *Agrobacterium* sp. for transformation

*Agrobacterium* sp. cultures were inoculated from stock on LB plates with appropriate selection and incubated at 28°C for 2 days. Liquid LB (25 ml) was inoculated with single colonies from plates. AGL-1_AO11 and EHA105_AO11 were cultured in medium containing 50 μg/ml kanamycin (10 μl stock / 10 ml medium). AGL-1 was grown with 25 μg/ml rifampicin (5 μl stock / 10 ml medium), and EHA105 with 15 μg/ml rifampicin (3 μl stock / 10 ml medium). Cultures were incubated at 28°C, 200 rpm, for ca. 22 h until reaching OD600 of 0.5–0.9.

*Agrobacterium* sp. AGL-1 or EHA105 pre-cultures (12–15 ml) were centrifuged 3500 rpm for 10 minutes, washed twice with 1 ml of freshly prepared liquid induction medium (IM) (S1 File, [47]), and resuspended in IM to an OD600 of ~0.3 in a total volume of ca. 25 ml. The culture was incubated at 28°C, 200 rpm, until OD600 doubled (0.6–0.8).

#### Preparation of fungal spore suspension

The *D*. *sapinea* spore suspension was prepared as described in Oostlander (2023) [30]. In brief *D*. *sapinea* spores were harvested from cultures (21 days incubation on VMM at 22°C and constant light) by rinsing with 0.01% Tween, counted and resuspended in IM to a concentration of 2 × 10^6^ spores/ml.

#### Co-cultivation

For co-cultivation, sterile nitrocellulose filters (MF-Millipore^™^ HAWP03700), cellophane, and filter paper were placed on freshly prepared 5.5 cm plates containing 5 ml of solid IM. The spore suspension was mixed with the bacterial suspension in ratios of 1:1, 1:10, or 10:1 to a total volume of 100 μl per transformation reaction, and 20 μl of IM was added. 110 μl of the mixture was pipetted onto the filter and spread on the filter by tilting the plate. After drying, the plates were incubated upside down for 24 h, 48 h or 72 h at temperature 20°C, 23°C or 26°C in darkness.

#### Selection

To select the transformed fungal cells and to eradicate the bacteria, the plates were overlaid with 5 ml selection medium (VMM + 6 μg/ml cefotaxim +10 μg/ml hygromycin B). The plates were incubated at 28°C for 20 days in darkness and checked for fungal growth daily after one week. Growing fungal colonies, assumed to be transformants, were selected and transferred to new selection plates (VMM + 6 μg/ml cefotaxim +10 μg/ml hygromycin B) for verification and further characterization.

#### Diagnostic PCR

Diagnostic PCRs were performed to verify the potential transformants. Using the oligonucleotides 2242/2243 (S1 Table), a segment of the T-DNA was amplified to confirm its presence in the fungal genome. Integration at the intended gene locus was assessed using oligonucleotide pairs 2244/2245 and 2246/2247 (S1 Table), wherein one oligonucleotide binds to the reporter gene cassette of the T-DNA, and the other oligonucleotides aligns outside the T-DNA in the genomic region flanking the *niaD* gene locus.

### Southern blot analysis

To determine the integration of the construct into the *D*. *sapinea* genome eight selected transformants were cultivated in 50 ml liquid VMM (2.5 μg/ml Hyg) for three days. Genomic DNA was isolated according to (Lee and Taylor 1990) and digested with *Hind*III. The DNA was separated on an agarose gel and transferred to a membrane (Amersham^™^ Hybond^®^ -XL). The probe utilized in this study was the T-DNA of the vector pAO-11 amplified by PCR with pAO-011 as the template DNA and primers 2082/2087 (S1 Table). Southern hybridization was performed following the instructions of the Roche DIG-High Prime DNA Labeling and Detection Starter Kit II (Cat. No. 11 585 614 910). Hybridization was carried out overnight at 62°C, followed by two 5-minute washing steps at room temperature with 2x SSC and 0.1% SDS, and then two 15-minute washing steps at 62°C with 0.5x SSC and 0.1% SDS.

### Hygromycin B sensitivity test

The sensitivity of the mutant as well as the WT strain to hygromycin B was tested on VMM with the addition of different concentrations of hygromycin B (5 μg/ml, 10 μg/ml, 20 μg/ml, 30 μg/ml, 40 μg/ml, 50 μg/ml and 100 μg/ml). Spores were harvested as described above and adjusted to a concentration of 1x10^5^ spores/ml in 0.01% Tween. The spore suspension (10 μl) was pipetted onto the test plates in three replicates. Fungal growth was assessed and documented 6 days after inoculation at room temperature and daylight using a Leica MZ6 stereomicroscope.

### Cultivation of *Pinus sylvestris* seedlings

Seeds of *Pinus sylvestris* provenance ASARUM FP-611 were purchased from Sveaskog Förvaltnings AB, (Stockholm Sweden). The seeds were soaked in sterile distilled water for 3 h prior to planting. Two or three seeds were sown in a pot of 6 cm diameter, filled with standard garden potting soil. Seedlings were kept at room temperature and daylight to germinate. Seedlings developed to around 4–6 cm in height and typically with five or six cotyledons. The seedlings were used for infection after 14 d.

### Infection on *Pinus sylvestris*

*Pinus sylvestris* seedlings (72 seedlings in total, 14 days old) were inoculated with WT spores (harvested from 21 days old cultures, as described above) in 6 treatment groups with about 12 plants per group (Table 2).

**Table 2:** Treatment groups for seedling infection.

group number	treatment	wounding	experimental group
1	1.5 μl spore suspension (1*10^5^ Spores in 0.05% Tween 20) on wound	Yes	treatment
2	1.5 μl 0.05% Tween 20 on wound	Yes	control
3	1.5 μl of a spore suspension (1*10^5^ Spores in 0.05% Tween 20)	No	treatment
4	1.5 μl 0.05% Tween 20	No	control
5	spore mass introduced into wound	Yes	treatment
6	wounding	Yes	control

For the first group, seedlings were inoculated by pipetting 1.5 μl of a spore suspension (1*10^5^ Spores in 0.05% Tween 20) onto a small wound introduced by pricking a cotyledon with a sterilized lancet (Carl Roth 6181.1, length 50 mm, wire-Ø 0.6 mm).

The second group was wounded with a sterilized lancet and 1.5 μl of a sterile 0.05% Tween 20 solution was pipetted onto the wounded cotyledon.

The third group was inoculated by pipetting 1.5 μl of a spore suspension (1*10^5^ spores/ml in 0.05% Tween 20) onto a healthy cotyledon.

The control group four was treated by pipetting 1.5 μl of a sterile 0.05% Tween 20 solution on the intact cotyledon.

The fifth group was inoculated by immersing an inoculation lancet into a spore mass released by a pycnidium of *D*. *sapinea*. Subsequently, the cotyledon was punctured using the inoculation lancet creating a minor incision through which the spores, which had adhered to the lancet, were effectively introduced into the wound site.

For the sixth group the cotyledon was punctured using a sterile lancet.

Development of symptoms was observed daily after inoculation for five days.

### Infection for microscopy

14 days old *P*. *sylvestris* seedlings were inoculated on the cotyledon and/or stem with spores of the *h2b-mcherry* expressing mutant strain (GD1_14). The seedlings were inoculated with the spore mass or a spore suspension (1*10^5^ spores/ml in 0.05% Tween 20) as described for the infection assay treatment 1 or 5.

### Microscopy

For live-cell-imaging of germlings and hyphae, VMM agar plates were inoculated with conidia suspended in 0.01% Tween 20 and incubated for 2–3 h at room temperature, until they germinated. For microscopy of hyphae, plates were inoculated and grown overnight at room temperature. Agar blocks of about 10 × 10 mm were cut from the cultures. The cultures were analyzed using light and fluorescence microscopy using a ZEISS Axio Observer Z1 microscope.

### *In-planta*-imaging

Samples for confocal scanning laser microscopy (CLSM) were transferred to a droplet of 20% glycerin (Carl Roth, Karlsruhe, Germany) on a microscope slide and covered with a cover slip. Fluorescence was recorded with a confocal laser scanning microscope (Leica TCS SP8, Leica, Wetzlar, Germany). Images were taken using the HC PL FLUOTAR 10x/0.32 DRY and the HC PLAPO CS2 20x/0.75 IMM objective and the Leica Application Suite X (LAS X) software for Leica microscopes. One hybrid detector (HyD, 610 nm—639 nm) and one photomultiplier tube (PMT2, 681 nm—698 nm) were used to capture the emission fluorescence of the mcherry-H2B construct—and the autofluorescence of plant and fungal tissue. Pseudocolours were used to represent specific emission fluorescence. Red pseudocolour (HyD1, 610 nm—639 nm) was used to visualize the mcherry labelled fungal nuclei. Green (PMT2, 681 nm—698 nm) pseudocolour was used to visualize plant autofluorescence. Multichannel images were produced by overlay of the two channels. Images were generated by maximum intensity projection of confocal z-stacks.

### Data analysis

Fisher’s exact test was used to compare different experiments. As a first step, replicates were tested for significant differences. All p-values for replicates were much larger than 0.05, indicating that the corresponding frequencies for the replicates did not differ significantly. Therefore, results from replicates were joined together and Fisher’s exact test was than applied to the joint replicates to compare different conditions.

## Results

### *Agrobacterium*-mediated-transformation of *D*. *sapinea*

**Fig 1:**
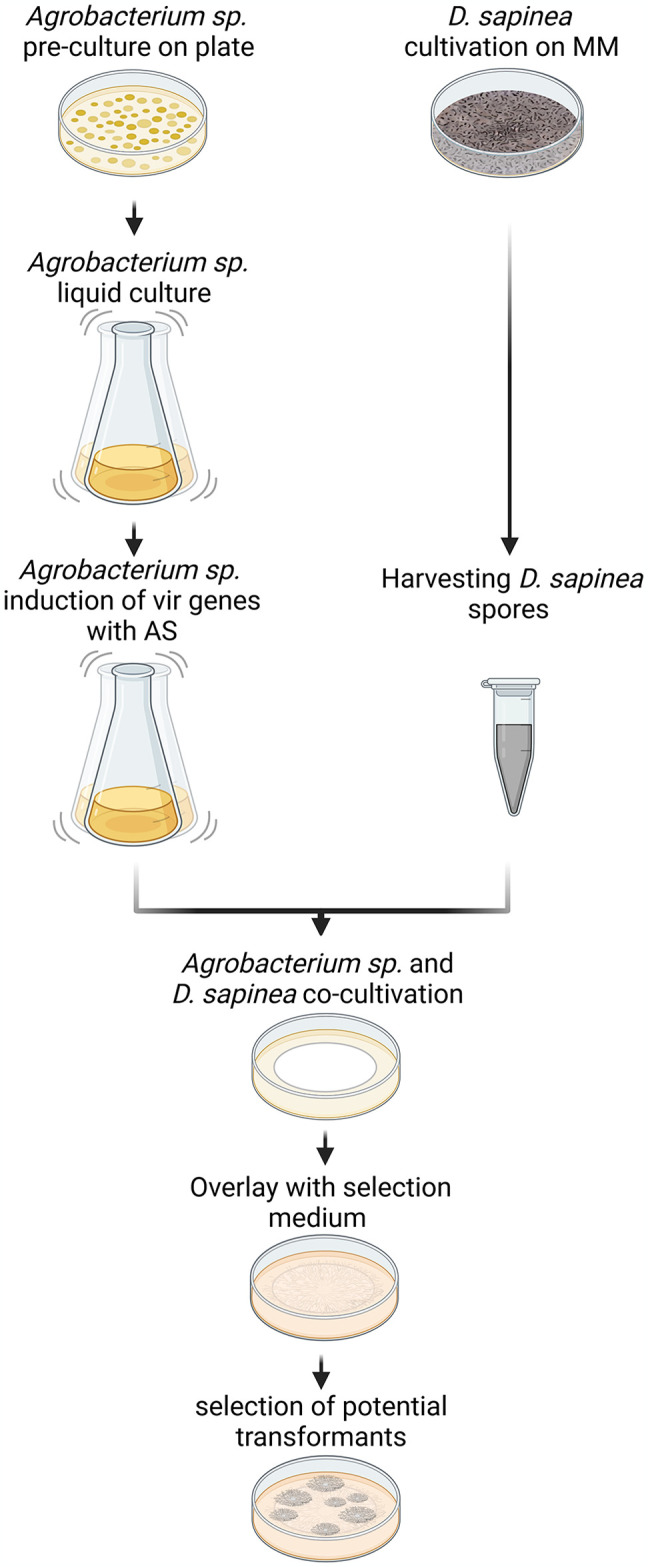
Schematic overview of *Agrobacterium*-mediated transformation of *D*. *sapinea*: *Agrobacterium* sp. ALG-1_AO11 and *D*. *sapinea* are initially cultured separately. After induction of the *vir* genes of *Agrobacterium* sp. ALG-1_AO11, the bacteria are co-cultured with *D*. *sapinea* spores. The plates are subsequently overlaid with antibiotic- and antifungal-containing medium, to eliminate the bacteria and to select the fungal transformants.

The goal of this study was to establish an *Agrobacterium*-mediated transformation protocol for the pine pathogen *D*. *sapinea* (Fig 1). As proof of principle, we chose to transform the fungus with an expression construct encoding histone H2B, tagged with mCherry, allowing rapid visual verification of obtained transformants. As an expression locus, we chose the *niaD* gene, which encodes a nitrate reductase. This gene locus is commonly used as an expression locus in filamentous fungi, since it is dispensable under standard growth conditions [48–50]. In the first step, the necessary *Agrobacterium* plasmid pAO-011 was constructed. This binary vector is based on plasmid pRFHUE-eGFP. It carries a T-DNA, that contains 1000 bp upstream and downstream of the *D*. *sapinea niaD* gene for homologous genome integration, a hygromycin B resistance cassette for selection of transformants and *h2b-mcherry* under the control of the *tef* promotor and *tef* terminator of *D*. *sapinea*.

To optimize a transformation protocol using this plasmid, we systematically varied and compared some of the known critical factors influencing *Agrobacterium*-mediated transformation. These parameters included: bacterial strain (AGL-1 and EHA105), ratio of spores to bacterial cells in the bacterium fungi co-cultivation (1:1, 10:1 und 1:10), co-cultivation duration (24 h, 48 h and 72 h) and temperature (20°C, 23°C and 26°C), and the substrate for co-cultivation (nitrocellulose filters, cellophane, and filter paper).

Plasmid pAO-011 was transformed into AGL-1 and EHA105, resulting in the strains AGL-1-AO011 and EHA105-AO011, respectively.

As the fungal starting material for the transformation asexual spores of *D*. *sapinea* strain CBS138184 were used in all experiments, which were produced according to our earlier established sporulation protocol [30]. Co-cultivations with *Agrobacterium* were conducted with the above-mentioned parameter variations. All other experimental factors remained consistent in all experiments according to standard protocols (S1 File).

Only co-cultivation of *D*. *sapinea* spores with strain AGL-1 at 20°C and 23°C for 72 h with a bacteria-spore-suspension ratio of 1:1 on nitrocellulose, resulted in the growth of putative transformants on the selection plates after approximately 20 days. The colonies were transferred to VMM with 10 μg/ml hygromycin B, where they exhibited robust growth, confirming the stable integration of the selection marker. The resulting transformation efficiency was 20 transformants per 1 x 10^5^ spores in two independent experiments. For quick and easy adaptation of this methodology, a "ready to use" laboratory protocol for *D*. *sapinea* transformation is provided in the S1 Movie.

#### *Agrobacterium*-mediated-transformation of *D*. *sapinea* leads to a high rate of homologous integration

**Fig 2:**
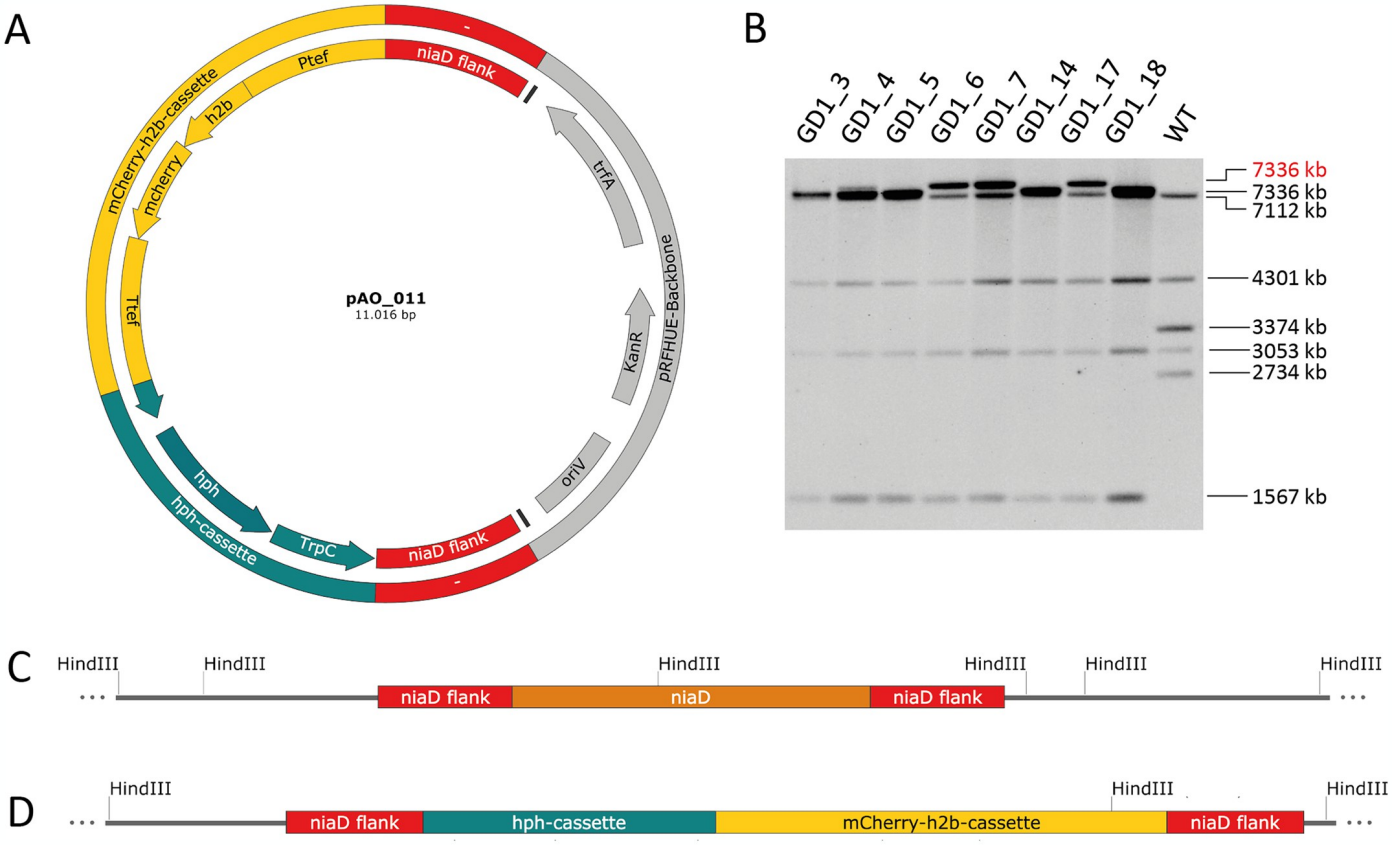
*Agrobacterium*-mediated transformation of *D*. *sapinea* (**a)** Schematic representation of the vector pAO-011. **(b)** Southern Blot analysis of 8 strains transformed with *Agrobacterium* sp. AGL-1_AO11. *Hind*III-digested DNA from transformants and the wild type (WT) was analyzed using the entire T-DNA fragment as a probe. **(c)** Schematic representation of the wild-type *niaD* locus. **(d)** Schematic representation of the *niaD* locus after homologous integration of the T-DNA of plasmid pAO-011.

After a successful transformation, fungi can integrate the introduced DNA into the genome through either heterologous or homologous integration. To facilitate homologous integration of the T-DNA, our constructed plasmid includes 1000 bp of the upstream and downstream flanks of the *D*. *sapinea niaD* homolog (Fig 2). This design allows for the substitution of the *niaD* gene with the *h2b-mcherry* expression cassette and the *hygR* resistance cassette. Diagnostic PCR confirmed that all transformants tested in this study integrated the expression cassette at the desired gene locus (S1 Raw Images).

Southern blot analysis was conducted to investigate whether these transformants had integrated unwanted additional transformation fragments at other genomic locations (Fig 2B). Hybridization of *Hind*III digested genomic wild-type DNA with the T-DNA fragment, containing the *niaD* flanking regions and the expression construct, as a probe, is expected to yield two bands of sizes 3374 bp and 2734 bp. Upon homologous insertion of the transformation fragment at this locus, two bands of sizes 7336 and 1567 are expected. This anticipated pattern was identified in five strains, while the remaining three strains exhibited an additional larger band, maybe caused by additional heterologous insertion or unexpected rearrangements at the *niaD* locus. Since these three strains did not show the desired pattern, they were excluded from further analysis.

#### The transformed *D*. *sapinea* strain shows reduced sensitivity to hygromycin B

For the first time, we used the *trpC* promoter and terminator to drive an antifungal resistance gene in *D*. *sapinea*. We evaluated the transformants’ resistance to hygromycin to assess the functionality of these components for heterologous expression, for future reference.

**Fig 3:**
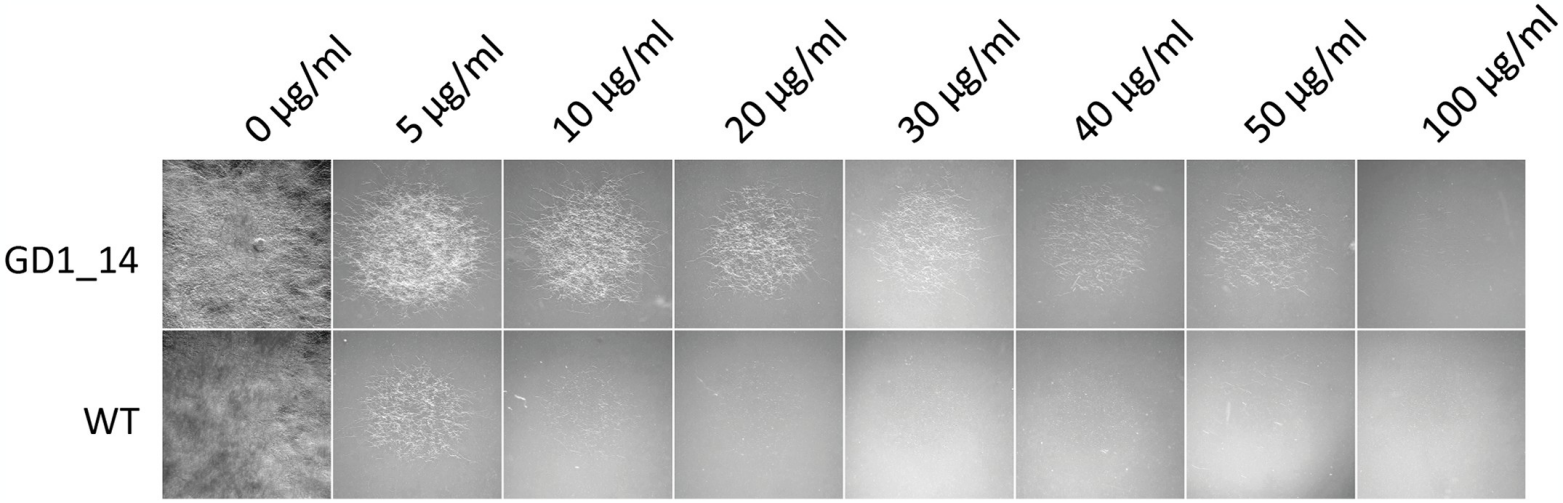
*D*. *sapinea* wild type (WT) and transformed strain (GD1_14) growing on VMM with increasing hygromycin B concentrations after six days of incubation.

Growth of the wild type and the mutant strain GD1_14 was evaluated on varying concentrations of hygromycin b (0, 5, 10, 20, 30, 40, 50, and 100 μg/ml) after six days of incubation. The wild type strain exhibited a strong reduction in growth even at the lowest concentration tested (5 μg/ml), and complete growth inhibition was observed at 30 μg/ml of hygromycin B (Fig 3). In contrast, the mutant strain still showed growth on a tenfold increased concentration of 50 μg/ml. At 100 μg/ml its growth was fully inhibited. These observations indicate the high suitability of the resistance gene and the promoter/terminator for heterologous expression in *D*. *sapinea*.

#### mCherry is suitable for subcellular protein localization in *D*. *sapinea*

**Fig 4:**
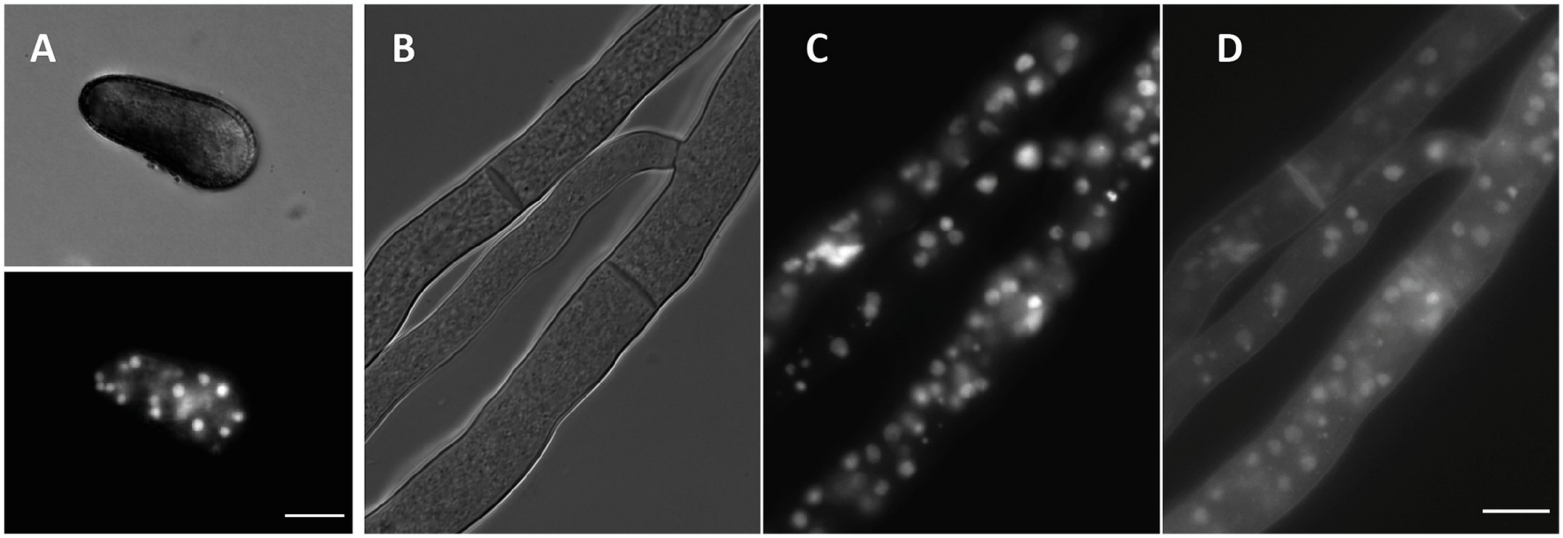
Microscopy of the transformed *D*. *sapinea* strain GD1_14 expressing the H2B-mcherry fusion protein on VMM. **(a)** Brightfield and fluorescence microscopy of a conidium. H2B-mcherry is detected by an excitation at 580 nm. **(b)** Brightfield microscopy of hyphae. **(c)** Fluorescence microscopy of hyphae detecting H2B-mcherry, excitation at 580 nm. **(d)** Fluorescence microscopy of hyphae stained with Hoechst 33258, excitation at 350 nm. Scale bars 10 μm.

A proof of principle expression construct contained the histone h2b gene fused to the red fluorescent protein mCherry. To test expression of this construct and the detectability of the fusion protein via the fluorescent tag, spores and hyphae of transformant GD1_14 were analyzed by fluorescence microscopy. Both cellular structures exhibited an intense fluorescence signal in punctate structures. To test if these signals co-localize with nuclei, hyphae were stained with the DNA-binding dye Hoechst 33258. This staining validated the successful labeling of nuclei with H2B-mCherry (Fig 4B). It was therefore confirmed that mCherry is a highly suitable marker for subcellular protein localization in *D*. *sapinea*. Notably, the mycelium displayed a high number of nuclei per compartment (Fig 4B and 4C and S1 Movie). Similarly, a substantial number of nuclei were evident in conidia (Fig 4D and 4E).

#### Development of a laboratory-adapted down-scaled *Pinus sylvestris* infection assay

**Fig 5:**
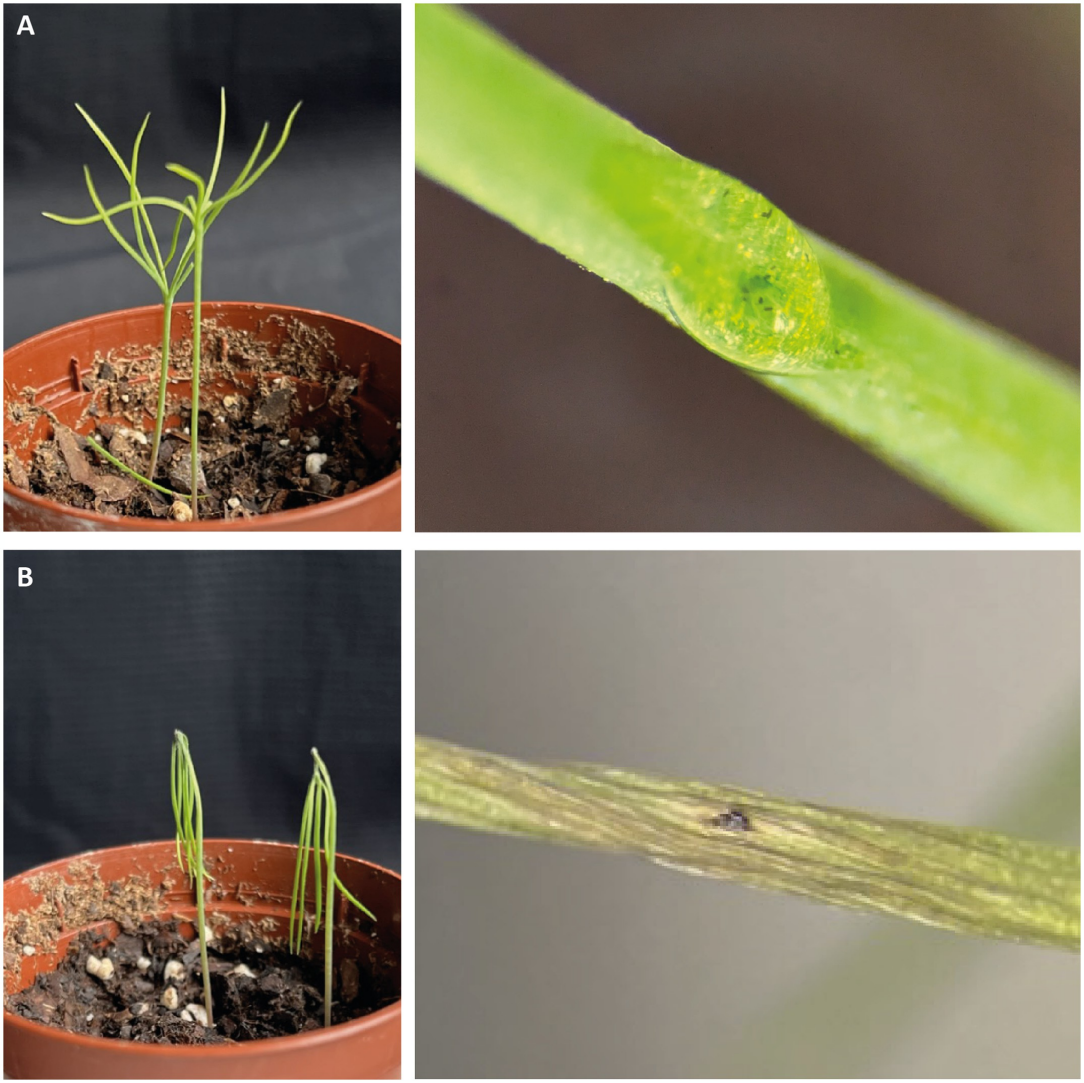
Representative pictures of 14 days old *P*. *sylvestris* seedlings infected with wild-type *D*. *sapinea* spores, and a close up of the inoculation site at **(a)** the day of inoculation and **(b)** five-days post inoculation.

After establishing mCherry as a suitable fluorescent marker in *D*. *sapinea*, we set out to test if this marker can also be used to detect growth of the fungus *in planta*. In an earlier study, we reported a spore-based infection assay of Scots pine. However, that study employed two-year-old pine saplings, which require sufficient greenhouse space. To develop a more efficient method for analyzing *D*. *sapinea* pathogenicity in terms of time, space, and cost, we used two-week-old laboratory-grown *P*. *sylvestris* seedlings for infection assays. These seedlings were inoculated with *D*. *sapinea* conidia using different application methods. Firstly, spore suspensions in Tween20 were applied to both wounded and unwounded cotyledons. Secondly, spores were directly transferred from pycnidia to the seedlings using an inoculation lancet, which created a small wound on the plant surface during application. Plant health was assessed five days after inoculation using visible wilting as readout (Fig 5).

Highest frequency of wilting was observed when the seedlings were wounded and inoculated with a spore suspension (100%), and inoculation with a spore mass obtained directly from pycnidia resulted in similar high infection success (77–100%, Table 3). These observations differed significantly from the results obtained for the corresponding control groups, where wilting was rare (0–17%) (p < 0.0001). Non- wounded, but spore-inoculated seedlings showed very limited wilting (15%) that did not significantly differ from the corresponding control group (23%). As a conclusion, two-week-old pine seedlings are suitable as host plants in laboratory scale pathogenicity assays for *D*. *sapinea*. In addition, our findings highlight the importance of the inoculation method for the outcome of the fungus-plant interaction.

**Table 3:** Results of the comparative *P*. *sylvestris* seedling infection assays using *D*. *sapinea* spores.

group number	treatment	wounding	experimental group	Fraction of wilted seedlings rep a	fraction of wilted seedlings (%) rep b
1	spore suspension	yes	treatment	**92%** (12/13)	**100%** (14/14)
2	0.05% Tween 20	yes	control	**8%** (1/13)	**0%** (0/13)
3	spore suspension	no	treatment	**15%** (2/13)	**0%** (0/13)
4	0.05% Tween 20	no	control	**23%** (3/13)	**0%** (0/13)
5	spore mass	yes	treatment	**84%** (11/13)	**100%** (16/16)
6	wounding	yes	control	**0%** (0/12)	**17%** (2/12)

#### Fluorescence labeling of *D*. *sapinea* enables the visualization of the host pathogen interaction

The two methods described above, the laboratory-scale infection assay and the fluorescence labeling of the fungus, could now be combined to visualize the infection *in planta*. Two-week-old *P*. *sylvestris* seedlings were wounded and infected with spores of the mutant strain GD1_14. When producing the required conidia, we noticed that the mutant strain produced significantly smaller numbers of spores than the wild type, while the number of pycnidia formed per plate was normal.

**Fig 6:**
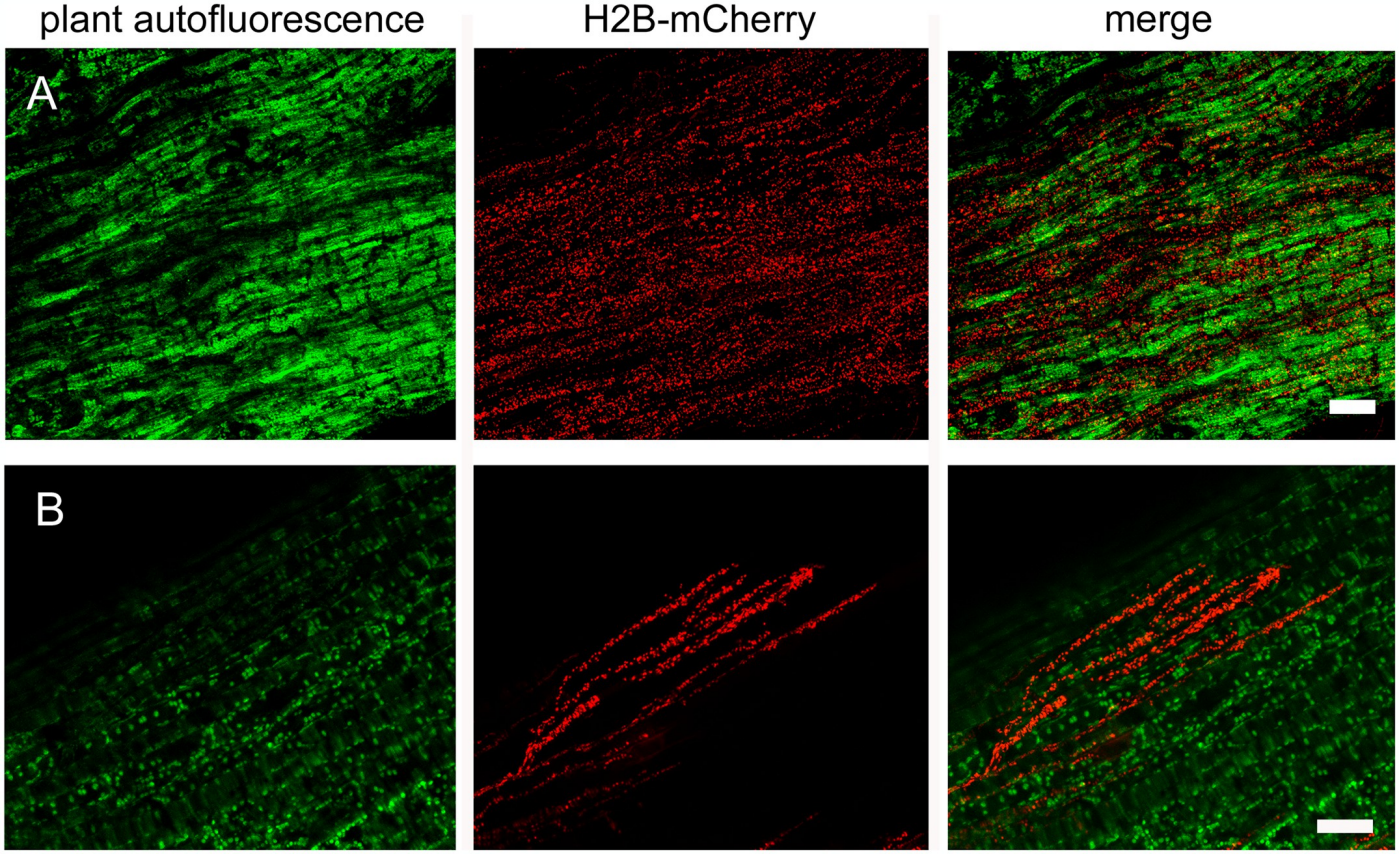
Confocal laser scanning micrographs of *P*. *sylvestri*s—*D*. *sapinea* (mCherry-tagged Histone H2B) interaction five days after the direct transfer of spores from pycnidia to a 14-day old seedling. **A** Seedling without visual symptoms. H2B-mcherry tagged hyphae of *D*. *sapinea* colonize the cotyledon. Maximum projection of 41 μm z-stack; **B** Seedling showing symptoms of infection. Maximum projection of 36 μm z-stack. Scale bar: 50 μm.

Plants were inoculated by the direct transfer of spores from pycnidia as described above. Two to five days after inoculation the seedlings exhibited initial signs of wilting and were examined using confocal laser scanning microscopy starting at the inoculation site. In plant tissue close to the inoculation site, which showed visually apparent signs of necrosis, heavy and dense colonization by the fungus was readily detected (Fig 6A). At the hyphal growth front, the plant material did not show signs of necrosis (Fig 6B). Mycelial growth in this area was primarily characterized by straight, branching hyphae found in between the plant cells, with seemingly little penetration or destruction of plant cells initially. These observations suggest a distinct progression in the interaction between the pathogen and the host plant, with initial phases marked by intercellular growth and minimal cellular degradation, followed by the onset of necrosis as the colonization intensified. This proof-of-concept infection study lays the groundwork for future detailed analyses, spanning from spore germination to full plant colonization.

## Discussion

This study provides for the first time a protocol for genetic transformation of *D*. *sapinea*. By applying this method, a fluorescent strain was created, expressing a mCherry-labeled histone H2B, proving that this fluorescent protein is a suitable marker for subcellular protein localization in this fungus. In addition, we developed a laboratory adapted space- and time-efficient method for analyzing the interaction of *D*. *sapinea* with its *P*. *sylvestris* host, employing small two-week-old seedlings. These advancements lay the groundwork for future studies ranging from molecular research to the cell biology of this plant-pathogen interaction.

The *Agrobacterium*-mediated transformation method used in this study is characterized by very simple handling and low costs compared to other transformation approaches and has the advantage of relying on standard laboratory equipment [34]. It is worth noting that attempts to achieve PEG-mediated protoplast transformation, which is commonly used in filamentous fungi, were not successful in the course of this study. Although protoplasts were obtained, no stable transformants could be isolated. The molecular bases of protoplast transformation are still not fully understood. The establishment of this method therefore largely relies on trial and error, often resulting in unsuccessful outcomes [32, 34].

In two *Botryosphaeriaceae* species, *B*. *dothidea* and *B*. *kuwatsukai*, protocols combining protoplasts with *Agrobacterium*-mediated transformation have been successfully established [37, 38]. Given the developed sporulation protocol for *D*. *sapinea*, we could bypass the need for protoplast production and directly utilize conidia for *Agrobacterium* co-cultivation. Exploring the adaptability of this protocol, including sporulation induction, to other *Botryosphaeriaceae* species could be a promising strategy.

The predominant mode of integration of DNA transformation fragments into the genome, either by heterologous or homologous recombination, is primarily dependent on the DNA repair strategy favored by the fungal species [51]. However, the rate of homologous recombination is often higher in *Agrobacterium*-mediated transformation compared to other methods [36]. In this study, the rate of homologous integration of the introduced DNA was high, rendering this protocol suitable for future gene targeting approaches, including gene inactivation or the targeted introduction of specific mutations. Southern blot analysis of the transformants suggested additional heterologous integrations or complex recombination events at the targeted gene locus in some isolates, which is a common observation in filamentous fungi [52, 53]. This finding highlights, however, the necessity for comprehensive molecular analysis of transformants prior to their use in extensive experimental studies.

Based on studies in the gray mold *B*. *cinerea* [48], we chose the *niaD* locus for the targeted integration of the expression construct. The *niaD* gene encodes the nitrate reductase, an enzyme that is non-essential under standard laboratory conditions for most fungi, provided that alternative nitrogen sources, such as ammonium salts, are readily available [48]. The deletion has no effect on the pathogenicity or general growth and development of *B*. *cinerea* and other plant pathogenic fungi [48–50]. The *D*. *sapinea* strain constructed in this study exhibits normal vegetative growth and produces numerous pycnidia on VMM. However, only few of these spore-bearing structures mature and release conidia. This reduced spore yield complicates obtaining adequate inoculum for extended infection studies and the mutation may also impact the infection process. A future challenge lies in identifying alternative gene loci for the integration of expression constructs.

In our proof-of-concept experiment using histone H2B to establish subcellular protein localization in *D*. *sapinea*, we observed intriguing traits of the mycelium and pycnidiospores. Specifically, both hyphal compartments and vegetative spores contained high numbers of nuclei. This highlights the importance of selecting strains post-transformation to obtain homokaryotic isolates, as only a fraction of the numerous nuclei integrates foreign DNA during the transformation process. This is of particular importance for the creation of gene knock-out mutants for gene function analysis. The very pronounced fluorescence observed in our strain also demonstrated that the *tef-1* promoter and terminator controlling the expression of the mCherry fusion construct, are excellently suited for protein localization studies or heterologous expression in *D*. *sapinea*.

To further simplify and standardize a method for studying the pathosystem *P*. *sylvestris*- *D*. *sapinea*, we analyzed inoculation of pine seedlings with pycnidiospores. The infection of two-week-old *P*. *sylvestris* seedlings was successful, targeting a life stage of the tree that had not been studied extensively previously. To the best of our knowledge, there are very few studies that utilize such young seedlings, and no study has employed *D*. *sapinea* spores for inoculation [54]. The utilization of seedlings this young has, until now, been mostly restricted to pathogenicity assays focused on root pathogens [55, 56]. The rapid manifestation of disease in young *Pinus* seedlings after spore inoculation opens avenues for developing high throughput virulence assays and presents an opportunity to compare infection dynamics among different isolates. This time- and space-efficient experimental setup is also of particular value when genetically modified strains are used, which are subject to specific regulatory requirements. The seedlings can be grown on the bench top at daylight or in a plant incubator. Moreover, infecting this young plant material proved highly practical for microscopy. The juvenile plant parts eliminate the necessity for complex sample preparation, such as histological sectioning, enabling direct visualization using a confocal laser microscope. However, infections at this stage of pine development may be subject to different mechanisms than infections of older plants. Therefore, the comparability of the results between different developmental stages cannot be assumed without experimental verification.

The *in planta* observations revealed some very beneficial features of using a fluorescently labeled strain for plant infection in the proof-of-concept experiment. The fluorescent nuclei facilitated clear visualization of infection in young seedlings, distinguishing between hyphae and plant material. Sample preparation was straightforward without the need for staining steps, and the distinct fluorescent marker confirmed infection by the intended pathogen rather than by a contaminant. Expression of the marker apparently did not interrupt the pathogenicity of the isolate, making it a suitable strain for more detailed future analysis of the infection process.

Until now, the sequence of colonization and symptom development by *D*. *sapinea*, as well as the nature of growth in symptom-free tissues, has remained unclear [57, 58]. In this initial study, we detected hyphal growth in non-necrotic tissue of symptomatically infected 14-day old seedlings, indicating that plant cells in the immediate vicinity of the hyphal growth front show no obvious damage during the early part of the infection. This observation needs to be confirmed through more in-depth studies on the progression of the infection, including older plants. Necrotic tissue exhibited an increasingly intense colonization over time, to a point where virtually no intact plant cells were evident and the host tissue was mostly replaced by fungal hyphae. Externally this tissue appeared crumpled and collapsed. These observations align with findings of other studies, where naturally infected trees [57], and two-year-old seedlings inoculated by mycelial agar plugs [59] were analyzed. Here, too, heavy colonization of symptomatic tissue by hyphae of *D*. *sapinea* was observed. However, neither study provided a description of hyphal growth in the plant tissue at the hyphal growth front.

In conclusion, the experimental toolbox developed in this study presents exciting opportunities for future research. Our laboratory-scale infection system simplifies the execution of transcriptomic, proteomic, and metabolomic experiments, while the transformation method and *in planta* fluorescence microscopy offer a platform for studying the function of key virulence-related genes. Future avenues include exploring correlations with infection processes in other life stages and extending the system to investigate tree seedlings in different tree pathogen systems. The system’s simplicity, efficiency, and versatility make it a valuable tool for advancing our understanding of fungal genetics and plant-pathogen interactions in Scots pine.

## Supporting information

S1 File Laboratory protocol for the *Agrobacterium*-mediated transformation of *Diplodia sapinea*. Accessible online at DOI: http://dx.doi.org/10.17504/protocols.io.5qpvok7ozl4o/v1.
(PDF)

S1 Table Oligonucleotides used in this study. (DOCX)

S1 Movie Video of merged brightfield and fluorescence microscopy of *D*. *sapinea* expressing the H2B-mcherry. Merged brightfield and fluorescence microscopy of the transformed *D*. *sapinea* strain GD1_14 on VMM, expressing the H2B-mcherry fusion protein. The H2B-mCherry fluorescence is triggered by excitation at 580 nm.
(AVI)

S1 Raw images Original images of diagnostic PCRs and Southern blot image supporting Fig 2. PCR analysis of eight transformed strains was performed using primer pairs (A) 2245/2246 and (B) 2247/2248 to confirm homologous integration of the expression cassette at the *niaD* gene locus. (C) Southern blot analysis of eight strains transformed with *Agrobacterium sp*. AGL-1_AO11 was performed using *Hind*III-digested DNA from transformants and wild type (WT), with the entire T-DNA fragment as a probe. The Thermo Scientific GeneRuler 1 kb DNA Ladder was used as a marker in all gels.
(PDF)
