# Supplementary material for: Development of a molecular genetics and cell biology toolbox for the filamentous fungus Diplodia sapinea
Source: PLoS One. 2024 Dec 27;19(12):e0308794. doi: 10.1371/journal.pone.0308794 (PMC11676576; doi:10.1371/journal.pone.0308794)
Supplement: S1 File — Accessible online at DOI: http://dx.doi.org/10.17504/protocols.io.5qpvok7ozl4o/v1. (PDF) [file pone.0308794.s001.pdf]

Aug 02, 2024

Agrobacterium-mediated transformation of Diplodia sapinea

DOI
dx.doi.org/10.17504/protocols.io.5qpvok7ozl4o/v1

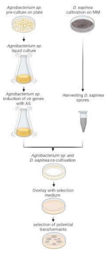

Anne Geertje Oostlander[1], Laura Brodde[2,3], Miriam von Bargen[1], Bernard Slippers[4], Yvonne Becker[5], Ulrike Brandt[1], Frank Klawonn[6], Christiaan Grobler[4], Lucas Well[1], Jan Stenlid[3], Jonàs Oliva[3,7], Malin Elfstrand[3], André Fleißner[1]

[1]Institute of Genetics, Technische Universität Braunschweig, Braunschweig, Germany;
[2]SCA Skog, NorrPlant, Sundsvall, Sweden;
[3]Department of Forest Mycology and Plant Pathology, Swedish University of Agricultural Sciences, Uppsala, Sweden;
[4]Department of Biochemistry, Genetics and Microbiology, Forestry and Agricultural Biotechnology Institute (FABI), University of Pretoria, South Africa;
[5]Institute for Epidemiology and Pathogen Diagnostics, Julius Kühn Institute (JKI)—Federal Research Centre for Cultivated Plants, Braunschweig, Germany;
[6]Helmholtz Centre for Infection Research (HZI), Braunschweig, Germany;
[7]Department of Agricultural and Forest Sciences and Engineering, University of Lleida, Lleida, Spain

Anne Geertje Oostlander
Technische Universität Braunschweig

OPEN ACCESS

DOI: dx.doi.org/10.17504/protocols.io.5qpvok7ozl4o/v1

Protocol Citation: Anne Geertje Oostlander, Laura Brodde, Miriam von Bargen, Bernard Slippers, Yvonne Becker, Ulrike Brandt, Frank Klawonn, Christiaan Grobler, Lucas Well, Jan Stenlid, Jonàs Oliva, Malin Elfstrand, André Fleißner 2024. Agrobacterium-mediated transformation of Diplodia sapinea. **protocols.io** https://dx.doi.org/10.17504/protocols.io.5qpvok7ozl4o/v1

License: This is an open access protocol distributed under the terms of the **Creative Commons Attribution License**, which permits unrestricted use, distribution, and reproduction in any medium, provided the original author and source are credited

Protocol status: Working
We use this protocol and it's working

Created: July 23, 2024

Last Modified: August 02, 2024

Protocol Integer ID: 103916

Keywords: Diplodia sapinea, Diplodia tip blight, Sphaeropsis sapinea, Agrobacterium-mediated-transformation, homologous integration, genetic transformation, ATMT

Funders Acknowledgement:
Marie Skłodowska-Curie grant
Grant ID: 101008129
Ellen and Tage Westins foundation
Grant ID: .

Abstract

This protocol details an *Agrobacterium*-mediated genetic transformation method for the fungal plant pathogen *Diplodia sapinea*. The technique results in high rates of homologous integration, enabling both targeted mutagenesis and heterologous gene expression.

Guidelines

Overview Workflow

	Transform *Agrobacterium* sp. AGL-1 and make glycerin-stocks	step 1
3 weeks before step 5	Inoculate plates with *D. sapinea* for spore production	step 5
Day 1	Plate *Agrobacterium* sp. AGL-1 from glycerin-stock and incubate	step 2
Day 4	*Agrobacterium* sp. AGL-1 Pre-culture	step 3
	Prepare liquid IM without AS and MES for step 4	see recipes
	Prepare IM-plates for step 6	see recipes
	Prepare fungal spore suspension	step 5
	Prepare selection plates for step 7	see recipes
Day 5	*Agrobacterium* sp. AGL-1 Main-culture	step 4
	Co-Cultivation	step 6
Day 8	Perform selection	step 7

Materials

Material.pdf

Potassium Buffer

Reference: Michielse et al. 2008

Ingredients:

- 1.25 M K_2HPO_4: 217.7 g K_2HPO_4
- 1.25 M KH_2PO_4: 170.1 g KH_2PO_4

Instructions:

- Fill each component up to 1 liter with water and autoclave.
- Add K_2HPO_4 solution to KH_2PO_4 solution until pH 4.8 is reached.

Magnesium-Sodium Solution

Reference: Michielse et al. 2008

Ingredients:

- 0.12 M $MgSO_4 \cdot 7H_2O$: 30 g $MgSO_4 \cdot 7H_2O$
- 0.25 M NaCl: 15 g NaCl

Instructions:

- Fill up to 1 liter with water and autoclave.

Calcium Chloride Solution

Reference: Michielse et al. 2008

Ingredients:

- 1% $CaCl_2$ (wt/vol): 10 g $CaCl_2 \cdot 2H_2O$

Instructions:

- Fill up to 1 liter with water and autoclave.

Glucose Solution

Reference: Michielse et al. 2008

Ingredients:

- 20% (wt/vol) $C_6H_{12}O_6$: 200 g $C_6H_{12}O_6 \cdot H_2O$

Instructions:

- Fill up to 1 liter with water and autoclave.

Ferric Sulfate Solution

Reference: Michielse et al. 2008

Ingredients:

- 0.01% $FeSO_4$: 0.1 g $FeSO_4 \cdot 7H_2O$

Instructions:

- Fill up to 1 liter with water and sterile filtrate.

Trace Elements

Reference: Michielse et al. 2008

Ingredients:

- 0.01% $ZnSO_4 \cdot 7H_2O$: 0.1 g $ZnSO_4 \cdot 7H_2O$
- 0.01% $CuSO_4 \cdot 5H_2O$: 0.1 g $CuSO_4 \cdot 5H_2O$
- 0.01% H_3BO_3: 0.1 g H_3BO_3
- 0.01% $MnSO_4 \cdot H_2O$: 0.1 g $MnSO_4 \cdot H_2O$
- 0.01% $Na_2MoO_4 \cdot 2H_2O$: 0.1 g $Na_2MoO_4 \cdot 2H_2O$

Instructions:

- Fill up to 1 liter with water and autoclave.

Ammonium Nitrate Solution

Reference: Michielse et al. 2008

Ingredients:

- 2.5 M NH_4NO_3: 200 g NH_4NO_3

Instructions:

- Fill up to 1 liter with water and sterile filtrate.

Solid Lysogeny Broth Medium (LB)

Reference: Bertani 1951

Ingredients:

- 5 g Yeast Extract
- 10 g Peptone
- 10 g Sodium Chloride
- 15 g Agar

Instructions:

- Fill up to 1 liter with water and autoclave.

Liquid Lysogeny Broth Medium (LB)

Reference: Bertani 1951

Ingredients:

- 5 g Yeast Extract
- 10 g Peptone
- 10 g Sodium Chloride

Instructions:

- Fill up to 1 liter with water and autoclave.

MES Buffer

Reference: Michielse et al. 2008

Ingredients:

- 1 M 2-(N-Morpholino) ethanesulfonic acid (MES): 195.24 g MES

Instructions:

- Adjust pH to 5.5 with NaOH.
- Fill up to 1 liter with water and sterile filtrate.
- Aliquot (10 ml) and store at -20°C in darkness.

Glycerol Solution

Reference: Michielse et al. 2008

Ingredients:

- 50% Glycerol (v/v): 500 ml Glycerol

Instructions:

- Fill up to 1 liter with water and autoclave.

Acetosyringone (AS) Solution

Reference: Michielse et al. 2008

Ingredients:

- 0.2 M Acetosyringone: 785 mg Acetosyringone

Instructions:

- Fill up to 20 ml with DMSO and sterile filtrate.
- Aliquot and store at –20°C in darkness.
- Concentration of work: 200 µM.

Hygromycin B Stock Solution

Ingredients:

- Hygromycin B (100 mg/ml): 1 g

Instructions:

- Fill up to 10 ml with dH_2O and sterile filtrate.
- Aliquot and store at –20°C in darkness.

Cefotaxime Stock Solution

Reference: Michielse et al. 2008

Ingredients:

- 0.2 M Cefotaxime: 955 mg Cefotaxime

Instructions:

- Fill up to 10 ml with dH_2O and sterile filtrate.
- Aliquot and store at –20°C in darkness.

Solid Induction Medium (IM)

Reference: Michielse et al. 2008

Ingredients:

- 15 g Agar
- 905.7 ml Water

Instructions:

- Autoclave.
- Directly before use, add for 1 l of medium:
- 800 µl Potassium buffer
- 20 ml Magnesium-sodium solution
- 1 ml Calcium chloride solution
- 5 ml Glucose solution
- 10 ml Ferric sulfate solution
- 5 ml Trace elements
- 2.5 ml Ammonium nitrate solution
- 10 ml Glycerol solution
- 200 µM Acetosyringone (AS) solution: 250 µl
- 40 ml MES buffer: 250 µl

Liquid Induction Medium (IM)

Reference: Michielse et al. 2008

Ingredients:

- 800 µl Potassium buffer
- 20 ml Magnesium-sodium solution
- 1 ml Calcium chloride solution
- 10 ml Glucose solution
- 10 ml Ferric sulfate solution
- 5 ml Trace elements
- 2.5 ml Ammonium nitrate solution
- 10 ml Glycerol solution
- 900.7 ml autoclaved water

Instructions:

- Directly before use, add for 1 l of medium:
- 200 µM Acetosyringone (AS): 1 ml AS
- 40 ml MES buffer

Vogels Trace Element Solution

Reference: Vogel 1956

Ingredients:

- 238 mM Citric acid (monohydrate): 5 g Citric acid (monohydrate)
- 174 mM $ZnSO_4 \cdot 7H_2O$: 5 g $ZnSO_4 \cdot 7H_2O$
- 25 mM $(NH_4)_2Fe(SO_4)_2 \cdot 6H_2O$: 1 g $(NH_4)_2Fe(SO_4)_2 \cdot 6H_2O$
- 10 mM $CuSO_4 \cdot 5H_2O$: 0.25 g $CuSO_4 \cdot 5H_2O$
- 3 mM $MnSO_4 \cdot H_2O$: 0.05 g $MnSO_4 \cdot H_2O$
- 8 mM H_3BO_3: 0.05 g H_3BO_3
- 2 mM $Na_2MoO_4 \cdot 2H_2O$: 0.05 g $Na_2MoO_4 \cdot 2H_2O$

Instructions:

- Dissolve all components successively in 95 ml distilled water while stirring at room temperature.

Vogels Biotin Solution

Reference: Vogel 1956

Ingredients:

- 5 mg Biotin

Instructions:

- Dissolve in 100 ml 50% ethanol.

Vogels Salts Solution

Reference: Vogel 1956

Ingredients:

- 125 g $Na_3Citrate \cdot 2H_2O$
- 250 g KH_2PO_4
- 100 g NH_4NO_3

- 10 g $MgSO_4 \cdot 7H_2O$
- 5 g $CaCl_2 \cdot 2H_2O$
- 5 ml Vogels Trace Element Solution
- 2.5 ml Vogels Biotin Solution

Instructions:

- Dissolve all components successively while stirring.
- Fill up to 1 liter with water.

Vogels Minimal Medium (VMM)

Reference: Vogel 1956

Ingredients:

- 20 ml Vogels Salts Solution
- 20 g Sucrose
- 15 g Agar

Instructions:

- Fill up to 1 liter with water and autoclave.

Transformation of electrocompetent *Agrobacterium* sp. AGL-1cells with plasmid DNA by electroporation

1 Thaw electrocompetent cells on ice.

2 Add 1 - 1.5 µl of plasmid DNA to 50 µl of cells.

3 Incubate on ice for 2 min.

4 Transfer the cell-DNA mixture to a chilled electroporation cuvette (2 mm) without introducing bubbles. Flick the cuvette downward quickly to distribute cells across the bottom of the well.

5 Electroporate the mixture with the following settings:
Voltage: 2500 V
Capacitance: 25 µF
Resistance: 400 Ω

6 Add 1 ml of LB medium to the cuvette immediately after pulsing and gently pipette up and down to resuspend the cells.

7 Transfer the cell suspension to a reagent tube and incubate the culture:
28 – 30°C
250 rpm
3 h

8 Spread the cells (10 µl, 100 µl, rest) onto selective plates with following selection markers:
50 µg/ml Kanamycin (10 µl stock / 10 ml medium)
25 µg/ml Rifampicin (5 µl stock / 10 ml medium)
100 µg/ml Carbenicillin (10 µl stock / 10 ml medium)

9 Incubate at 28 – 30°C. Transformed colonies are visible after 24-72 h.

10 Prepare a glycerin stock from several colonies and check via colony-PCR or plasmid preparation and PCR if your strains contain the expected fragment.

Plate *Agrobacterium* sp. AGL-1

11 Inoculate LB plates (10 ml) containing the following selection markers with *Agrobacterium* sp. AGL-1 from the glycerin stock. Use the untransformed strain as a negative control. Incubate at 28°C for about 2 days.
AGL1 transformed: 50 µg/ml Kanamycin (10 µl stock / 10 ml medium), 25 µg/ml Rifampicin (5 µl stock / 10 ml medium), 100 µg/ml Carbenicillin (10 µl stock / 10 ml medium)

AGL1 untransformed: 25 µg/ml Rifampicin (5 µl stock / 10 ml medium), 100 µg/ml Carbenicillin (10 µl stock / 10 ml medium)

Pre-culture of *Agrobacterium* sp. AGL-1

12 Inoculate 25 ml of liquid LB with the following selection markers in a 250 ml flask with a colony from fresh plates:
AGL1 transformed: 50 µg/ml Kanamycin (25 µl stock / 25 ml medium)

AGL1 untransformed: 25 µg/ml Rifampicin (12,5 µl stock / 25 ml medium)

13 Incubate until the cultures reached an OD_{600nm} of 0.5 to 0.9:
200 rpm
28°C
~ 22 h

Main-culture *Agrobacterium* sp. AGL-1

14
Transfer 12-15 ml of the Agrobacterium sp. AGL-1 suspension to a 50 ml centrifuge tube and centrifuge at:
3500 rpm
10 min

15 Wash the pellet with 1 ml freshly made liquid IM (see table, 100 ml Medium + 100 µl AS + 4 ml MES buffer) and centrifuge at:
3500 rpm
10 min

16 Resuspend the pellet in liquid IM to an OD_{600nm} of about 0.3.

17 Incubate ca. 25 ml until the OD_{600} is doubled (about 0.6 – 0.8) in a 250 ml Erlenmeyer flask.
28°C
200 rpm
8 - 10 h

Preparation of fungal spore suspension

18 Harvest spores of *D. sapinea* by rinsing the plate with 0.01 % Tween (Incubated on VMM, 21 d, constant light, 5000 – 7000 Lux)

19 Centrifuge spores for 10 s at 5000 rpm, discard supernatant.

20 Wash spores twice with 1 - 2 ml liquid IM and centrifuge at:
5000 rpm
10 s

21 Resuspend cells in IM to a concentration of $2 \cdot 10^6$ spores/ml. 50 µl suspension is needed per transformation.

Co-Cultivation

22 Onto 5.5 cm IM plates (freshly made or made the day before, ca. 5 ml medium per plate; stored in darkness at 4°C) place a nitrocellulose filter (MF-Millipore™ HAWP03700) with sterile tweezers.

23 Mix 50 µl of the spore suspension and 50 µl of the *Agrobacterium* sp. AGL-1 culture and 20 µl IM per transformation.

24 Pipette 110 µl of the mixture onto the filter and spread by tilting the plate. Ensure that the suspension does not run off the filter.

25 Incubate at:
22°C
upside down
in darkness
3 days

Selection

26 Add ca. 5 ml selection medium per plate (freshly made or made a few days before).
Selection medium:
VMM + 300 µM Cefotaxim (150 µl in 50 ml) +10 µg/ml Hygromycin B (10 µl in 100 ml)

27 Incubate at:
28°C
1 - 2 weeks
darkness

Harvest of Transformants

28 Pick fungal colonies that grow through the selection medium and transfer them onto new selection plates (VMM + 10 µg/ml Hygromycin B (10 µl in 100 ml)). Verify successful transformation by PCR.

Protocol references

Michielse CB, Hooykaas PJJ, van den Hondel CAMJJ, Ram AFJ. Agrobacterium-mediated transformation of the filamentous fungus Aspergillus awamori. Nat Protoc 2008; 3(10):1671–8.

Vogel HJ. A convenient growth medium for *Neurospora crassa* (Medium N). Microbial genetics bulletin 1956; (13):42–3.
