# Supplementary material for: Development of a molecular genetics and cell biology toolbox for the filamentous fungus Diplodia sapinea
Source: PLoS One. 2024 Dec 27;19(12):e0308794. doi: 10.1371/journal.pone.0308794 (PMC11676576; doi:10.1371/journal.pone.0308794)
Supplement: S1 Table — (DOCX) [file pone.0308794.s002.docx]

Table S1: Oligonucleotides used in this study

| **Number** | **Sequence (5’ -> 3’)** | **Purpose** |
| --- | --- | --- |
| 2080 | GTCGCCGCACTACATTAAAAACGTCCGC | Amplification of pRFHUE-eGFP backbone |
| 2081 | GGTGGCGGAGGCTCATGATCAGATTGTC | Amplification of pRFHUE-eGFP backbone |
| 2084 | AGCGTACATGGAGGATCCTCTAGAAAGAAG | Amplification of *hph* |
| 2085 | ACAAATGTGATCGACAGAAGATGATATTG | Amplification of *hph* |
| 2094 | TGCTCACCATTTTGGTGGAGGAAGAGTACTTGG | Amplification of *h2b* |
| 2095 | CACCGTCACCATGCCCCCTAAGGCCCAG | Amplification of *h2b* |
| 2096 | TAGGGGGCATGGTGACGGTGTGGAAGTG | Amplification of *Ptef* |
| 2097 | CCTTTGAACAACAAATGTGAACATGTAGATCAGTAAATCATGGC | Amplification of *Ptef* |
| 2090 | TCAATATCATCTTCTGTCGATGGTGGTCTGGACGCAAAATG | Amplification of T*tef* |
| 2091 | GTACAAGTAGGTAAGCAACACTCCCTTCCAATATAC | Amplification of T*tef* |
| 2082 | GATCATGAGCCTCCGCCACCACTCAACG | Amplification of *niaD* 5' flank |
| 2083 | GAGGATCCTCCATGTACGCTTCCGCCGATG | Amplification of *niaD* 5' flank |
| 2086 | CTTCTGTCGATCACATTTGTTGTTCAAAGGCAAAAGCTCG | Amplification of *niaD* 3' flank |
| 2087 | TTTTAATGTAGTGCGGCGACAGCGAAGC | Amplification of *niaD* 3' flank |
| 2088 | TCACATTTGTTGTTCAAAGG | Amplification of pAO-010 fragment |
| 2089 | TCGACAGAAGATGATATTG | Amplification of pAO-010 fragment |
| 2242 | CAACGTGACACCCTGTGCAC | Diagnostic PCR T-DNA |
| 2243 | GAAGCTGCGCGGCAC | Diagnostic PCR T-DNA |
| 2244 | AGGCGGCTGCGTCAAA | Diagnostic PCR – homologous integration at 3' |
| 2245 | GCCAATGCGGCGTGATATCG | Diagnostic PCR – homologous integration at 3' |
| 2246 | TGCGCCCGGACTGT | Diagnostic PCR – homologous integration at 5' |
| 2247 | CTTGACTATGAAAATTCCGTCACCAGC | Diagnostic PCR – homologous integration at 5' |
