# Supplementary material for: Development of a molecular genetics and cell biology toolbox for the filamentous fungus Diplodia sapinea
Source: PLoS One. 2024 Dec 27;19(12):e0308794. doi: 10.1371/journal.pone.0308794 (PMC11676576; doi:10.1371/journal.pone.0308794)
Supplement: S1 Raw images — PCR analysis of eight transformed strains was performed using primer pairs (A) 2245/2246 and (B) 2247/2248 to confirm homologous integration of the expression cassette at the niaD gene locus. (C) Southern blot analysis of eight strains transformed with Agrobacterium sp. AGL-1_AO11 was performed using HindIII-digested DNA from transformants and wild type (WT), with the entire T-DNA fragment as a probe. The Thermo Scientific GeneRuler 1 kb DNA Ladder was used as a marker in all gels. (PDF) [file pone.0308794.s004.pdf]

A

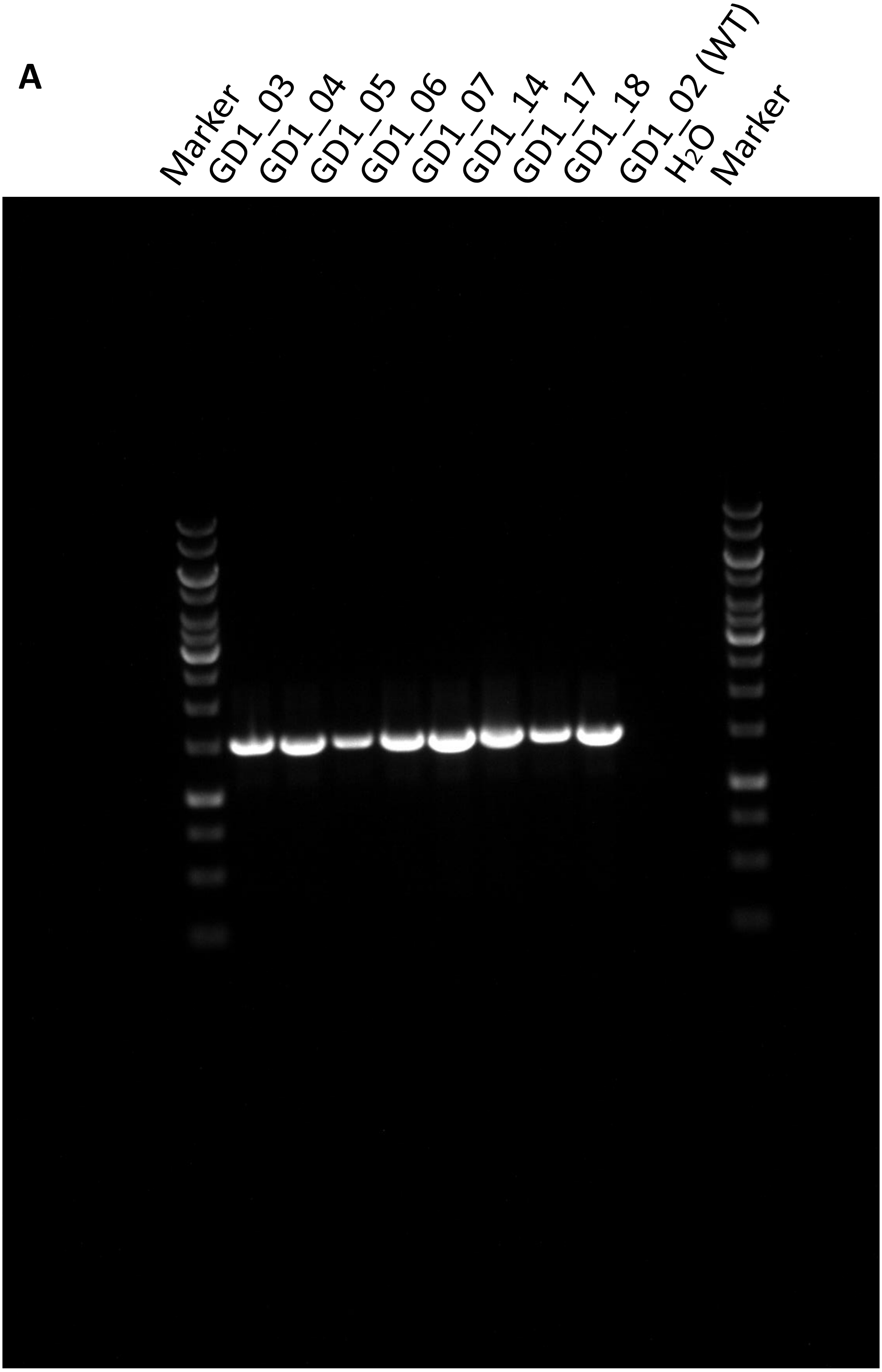
Marker
GD1_03
GD1_04
GD1_05
GD1_06
GD1_07
GD1_14
GD1_17
GD1_18
GD1_02 (WT)
H_2O
Marker

B

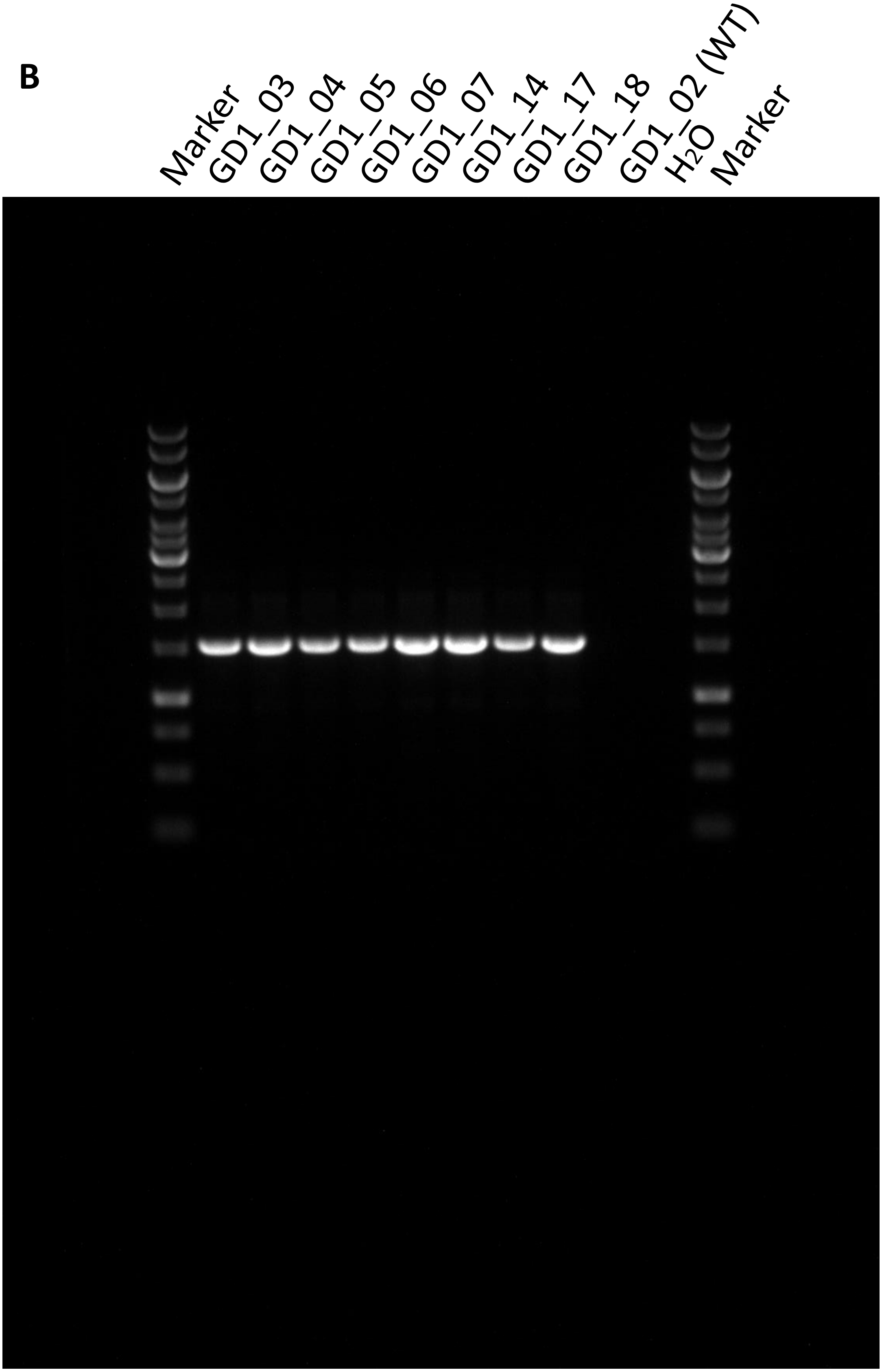

Marker
GD1_03
GD1_04
GD1_05
GD1_06
GD1_07
GD1_14
GD1_17
GD1_18
GD1_02 (WT)
H2O
Marker

C

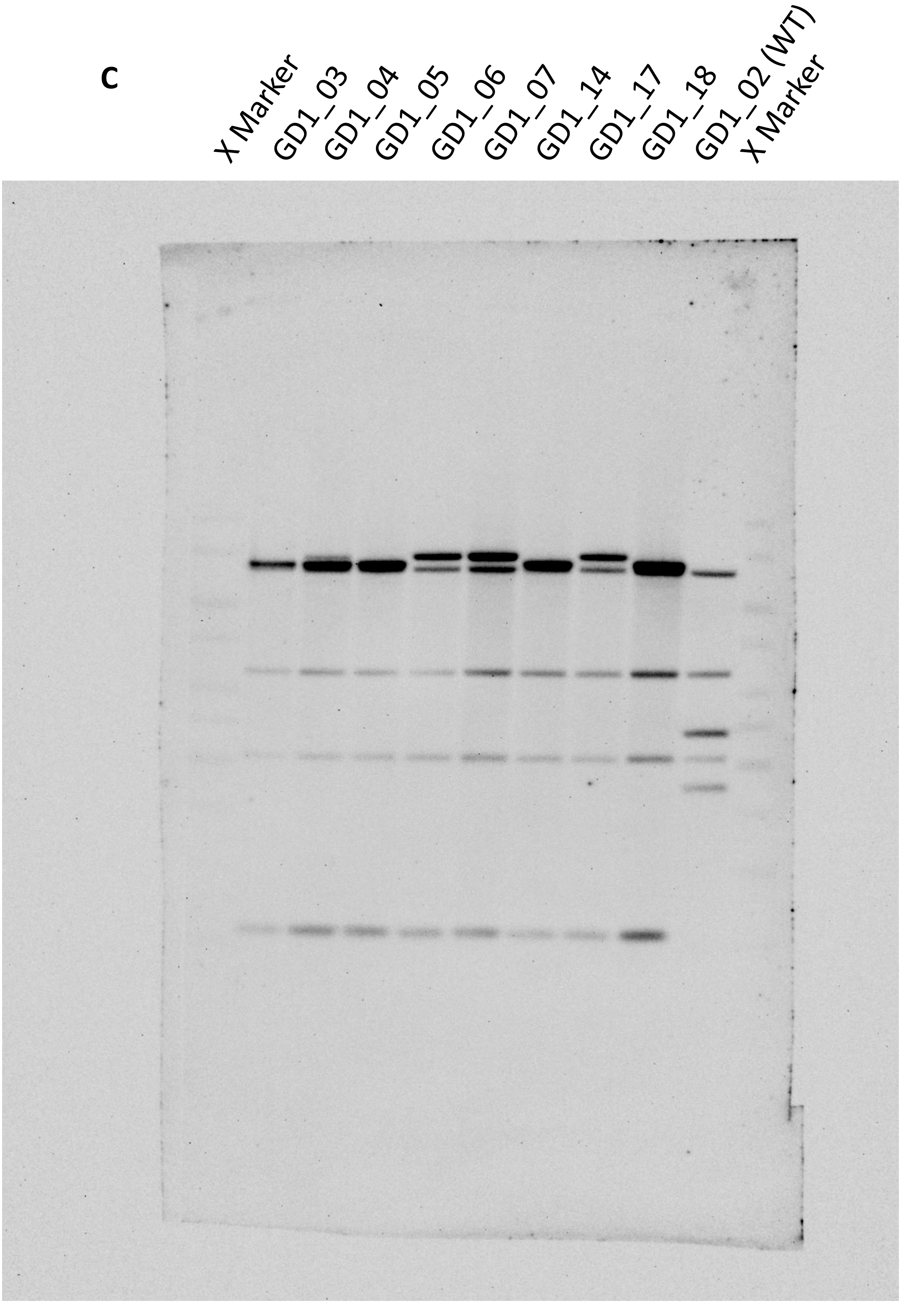
X Marker
GD1_03
GD1_04
GD1_05
GD1_06
GD1_07
GD1_14
GD1_17
GD1_18
GD1_02 (WT)
X Marker
